# Response inhibition alterations in migraine: evidence from event-related potentials and evoked oscillations

**DOI:** 10.1186/s10194-020-01187-2

**Published:** 2020-10-02

**Authors:** Guoliang Chen, Yansong Li, Zhao Dong, Rongfei Wang, Dengfa Zhao, Ignacio Obeso, Shengyuan Yu

**Affiliations:** 1 Medical School of Chinese PLA, Beijing, China; 2 Department of Neurology, The first Medical Center, Chinese PLA General Hospital, Fuxing Road 28, Haidian District, Beijing, 100853 China; 3 Department of Psychiatry, The 967th Hospital of Joint Logistic Support Force of PLA, Dalian, China; 4 Reward, Competition and Social Neuroscience Lab, Department of Psychology, School of Social and Behavioral Sciences, 210023 Nanjing, China; 5 Institute for Brain Sciences, Nanjing University, 210023 Nanjing, China; 6 HM Hospitales – Centro Integral en Neurociencias HM CINAC, Móstoles, Madrid, Spain

**Keywords:** Migraine, Response inhibition, ERPs, N2, P3, Theta oscillation, Delta oscillation, Cortical disexcitability

## Abstract

**Background:**

Migraine is characterized by a hypersensitivity to environmental stimulation which climaxes during headache attacks but persists during attack-free period. Despite ongoing debates about the nature of the mechanisms giving rise to this abnormality, the presence of deficient inhibitory cortical processes has been proposed to be one possible mechanism underlying its pathogenesis. Empirical evidence supporting this claim is mainly based on previous accounts showing functional cortical disexcitability in the sensory domain. Considering that a general inhibitory control process can play an important role across early to later stage of information processing, this may indicate the important role other dimensions of inhibitory control can play in migraine disability. The present study examined the pathophysiological features of inhibitory control that takes place during suppression of prepotent responses in migraineurs.

**Methods:**

Twenty-two patients with migraine without aura (mean age = 30.86 ± 5.69 years; 19 females) during the interictal period and 25 healthy controls (mean age = 30.24 ± 3.52 years; 18 females) were recruited. We used a stop signal task in combination with event-related potentials (ERPs) to examine participants’ neural activity supporting response inhibition.

**Results:**

Behaviorally, migraineurs exhibited prolonged stop signal reaction times relative to healthy controls. At the neural level, the amplitude of the stop-N2 over fronto-central, central and centro-parietal scalp regions, a component of the ERPs related to conflict monitoring during early, non-motoric stages of inhibition, was significantly increased in migraineurs. Meanwhile, the amplitude of the stop-P3 over central and centro-parietal scalp regions, a component of the ERPs reflecting late-stage inhibition of the motor system and cognitive evaluation of motor inhibition, was also significantly increased in migraineurs. Ultimately, our time-frequency analysis further revealed increased delta activity in migraineurs.

**Conclusions:**

Consistent with the theory that alterations in cognitive cortical processes are a key signature of migraine, our findings revealed an abnormal state of suppressing prepotent responses in migraineurs, which can be attributed to cortical disexcitability of the pre-frontal executive network and centro-parietal sensorimotor network. These novel findings extend to show the existence of dysfunctional inhibition control that occurs during suppression of prepotent responses in migraneurs.

## Introduction

Migraine is a common episodic neurological disorder mainly characterized by recurrent headache attacks, which has a detrimental influence on quality of life [1, 2]. The annual prevalence of migraine estimated worldwide is around 9%, which predominantly affects females [3–6]. Considerable empirical efforts have been devoted to understanding the causes of migraine, which would help with the detection of risk factors, the improvement of diagnosis and the development effective therapeutic interventions [7]. Although the exact pathogenetic mechanisms behind migraine are yet unclear, migraine is suggested to be a complex brain disorder with possible dysregulation between excitatory and inhibitory cortical imbalance [8, 9]. To enlarge our view on the neural deficits of the migraine brain, measuring neural function with electrophysiological methods (i.e., Electroencephalogram, EEG) can be an effective approach to enrich our view on the pathophysiology of migraine [10–13].

The majority of electrophysiological studies on stimulus processing in the visual, auditory, somatosensory and nociceptive domains strongly suggest that migraine is associated with a state of functional cortical disexcitability. Migraineurs display a lack of physiological habituation to repeated sensory stimulation especially during the intervals between attacks [10, 14, 15]. Despite ongoing debates about the nature of the mechanisms giving rise to these abnormalities [8, 16], the presence of deficient inhibitory cortical processes has been argued to be one possible mechanism underlying the pathogenesis of these conditions [16–20]. Available evidence supporting this claim is mainly based on previous studies on the cortical response to external sensory stimuli in migraineurs [10, 14, 21, 22]. For example, reliable differences in cortical disexcitability in response to sensory stimulation between migraineurs and healthy controls have been found, usually reflected by increased amplitude of evoked responses [17, 23, 24] and decreased activity of GABA-mediated inhibition of the sensory cortex [25]. Given that inhibition control does not take place only at the sensory level (such as inhibition of previously activated sensory processes), it can play an important role across early to later stage of information processing, such as inhibition of irrelevant information or an initiated response [26, 27]. This may draw our attention to the important role other dimensions of inhibitory control can play in migraine disability. Indeed, a few behavioral studies using the Stroop interference task have found an impairment of attentional control (inhibition of task-irrelevant information) in migraineurs during migraine attacks [28, 29], during the intervals between attacks [30] or during its chronicity [31]. This is strengthened by electrophysiological evidence showing increased amplitude of ERP components associated with Stroop interference in migraineurs during the intervals between attacks [32]. Despite such promising findings, it is surprising that knowledge of pathophysiological features underlying migraineurs’ abilities to suppress prepotent responses is still sparse.

Therefore, the present study was designed to examine the pathophysiological characteristics underlying suppression of behavioral responses by using a stop-signal task (SST) in combination with event-related potentials (ERPs) in patients with migraine without aura (MwoA) during the interictal period. The stop-signal task is a typical paradigm for measuring inhibitory control of an ongoing motor response [33, 34]. Participants are instructed to quickly respond to a primary Go stimulus while inhibiting their ongoing movement if a stop-signal stimulus is displayed. Considering that the promise of ERP-based biomarkers of cognitive dysfunction has been increasingly recognized in psychiatric [35, 36] and neurological disorders [37] including migraine [10, 12, 13], this technique can thus greatly help in examining how the brain detects the stop signal and decides to stop a prepotent motor response in migraineurs. Previous ERP studies have consistently identified two typical ERP components related to response inhibition in healthy participants: the stop-N2 and stop-P3 [38]. The stop-N2 refers to a negative wave that occurs 200–300 ms following a stop signal, with its maximum amplitude over frontocentral, central and centroparietal scalp regions. Meanwhile the stop-P3 is a large positive wave that peaks around 300–600 ms following a stop signal, with its maximum amplitude over central and centroparietal locations [38]. In spite of some debates about the functional significance of these two components, they are considered to reflect different sub-processes that underline response inhibition. Specifically, the stop-N2 is proposed to primarily reflect conflict monitoring during early, non-motoric stages of inhibition, while the stop-P3 is mainly thought to reflect late-stage inhibition of the motor system itself and cognitive evaluation of motor inhibition [38–41]. Given that common time-domain ERP measures may not accurately reflect multiple processes underlying response inhibition, the use of time–frequency (TF) analysis can provide complementary information about underlying processes behind the stop N2–P3 complex. Indeed, event -related theta (4–8 Hz) and delta (1–4 Hz) oscillations have been argued to index two separable, but highly overlapping processes underlying the stop N2–P3 complex during response inhibition, although their functional significance has yet to be fully clarified [38, 42]. Given that previous research has revealed deficits in the suppression of task-irrelevant information in terms of slower reaction times and increased amplitude of evoked responses in the Stroop interference condition in migraineurs [32], we can expect dysfunctional response inhibition (prolonged stop signal reaction times) and increased amplitude of both stop-N2 and stop-P3 in patients with MwoA. Moreover, time–frequency decomposition has been employed to characterize separable but overlapping processes underlying the stop N2–P3 complex in response inhibition in patients with MwoA.

## Methods

### Participants

In the present study, 22 right-handed patients with MwoA (age = 30.86 ± 5.69 years; 19 females) and 25 healthy controls (age = 30.24 ± 3.52 years; 18 females) were recruited. All participants had normal or corrected-to-normal vision. Both groups were matched in terms of sex, age and years of education. No history of neurological or psychiatric disorders and no migraine in first-degree relatives was reported by healthy controls. Patients with MwoA were screened with neurologic and physical evaluations by trained neurologists (Z.D. and S.Y.) as well as standard neuropsychological assessment by neuropsychologists (G.C.). The inclusion criteria for patients were: 1) fulfilling the diagnosed criteria for migraine without aura according to the International Classification of Headache Disorders, 3rd edition (ICHD-3); 2) at least 2 year’s history of migraine and at least one migraine episode per month and 3) outside migraine attacks during the experiment (the interictal period). Moreover, the following exclusion criteria were used: 1) neurological diseases (i.e., epilepsy, neuromuscular disorders); 2) mental retardation; 3) a current or past history of substance dependence, 4) receiving prophylactic anti-migraine therapy; 5) having suicide ideation and/or previous suicide attempts and 6) the presence of periodic limb movement disorder (i.e., nocturnal hyperkinesias) and recurrent parasomnias (> 3 episodes per week). All female participants from both groups took no oral contraceptives for at least 1 week. Demographic and clinical characteristics are described in Table 1.

**Table 1:** Demographic and clinical characteristics of the study sample

	MwoA (***n*** = 22) (M ± SEM)	Controls (***n*** = 25) (M ± SEM)	Group comparison
Age, years	30.86 ± 1.21	30.24 ± 0.70	t (45) = − 0.46, *p* > 0.05
Gender (F/M)	(19/3)	(18/7)	χ^2^ = 2.40, *p* > 0.05
Education, years	15.55 ± 0.59	15.32 ± 0.45	t (45) = −0.31, *p* > 0.05
BMI [kg/m^2^]	21.09 ± 0.77	21.04 ± 0.52	t (45) = − 0.06, *p* > 0.05
SAS	44.43 ± 2.27	39.45 ± 1.49	t (45) = − 1.88, *p* > 0.05
SDS	44.67 ± 2.97	42.85 ± 2.13	t (45) = − 0.51, *p* > 0.05
Duration of migraine, hours	30.61 ± 5.47		
History of migraine, years	12.41 ± 1.34		
Migraine frequency, times per month	5.00 ± 0.92		
Severity of headache (VAS scale)	8.23 ± 0.25		

*VAS* visual analog scale, with 0 indicating no pain and 10 worst possible pain, *SAS* Self-Rating Anxiety Scale, *SDS* Self-Rating Depression Scale, *BMI* body mass index, *M* mean, *SEM* standard error of the mean

All participants volunteered to participate in the present study. They all signed consent forms and the Ethics Committee of the Chinese PLA General Hospital approved the study protocol.

### Stop-signal task

**Fig. 1:**
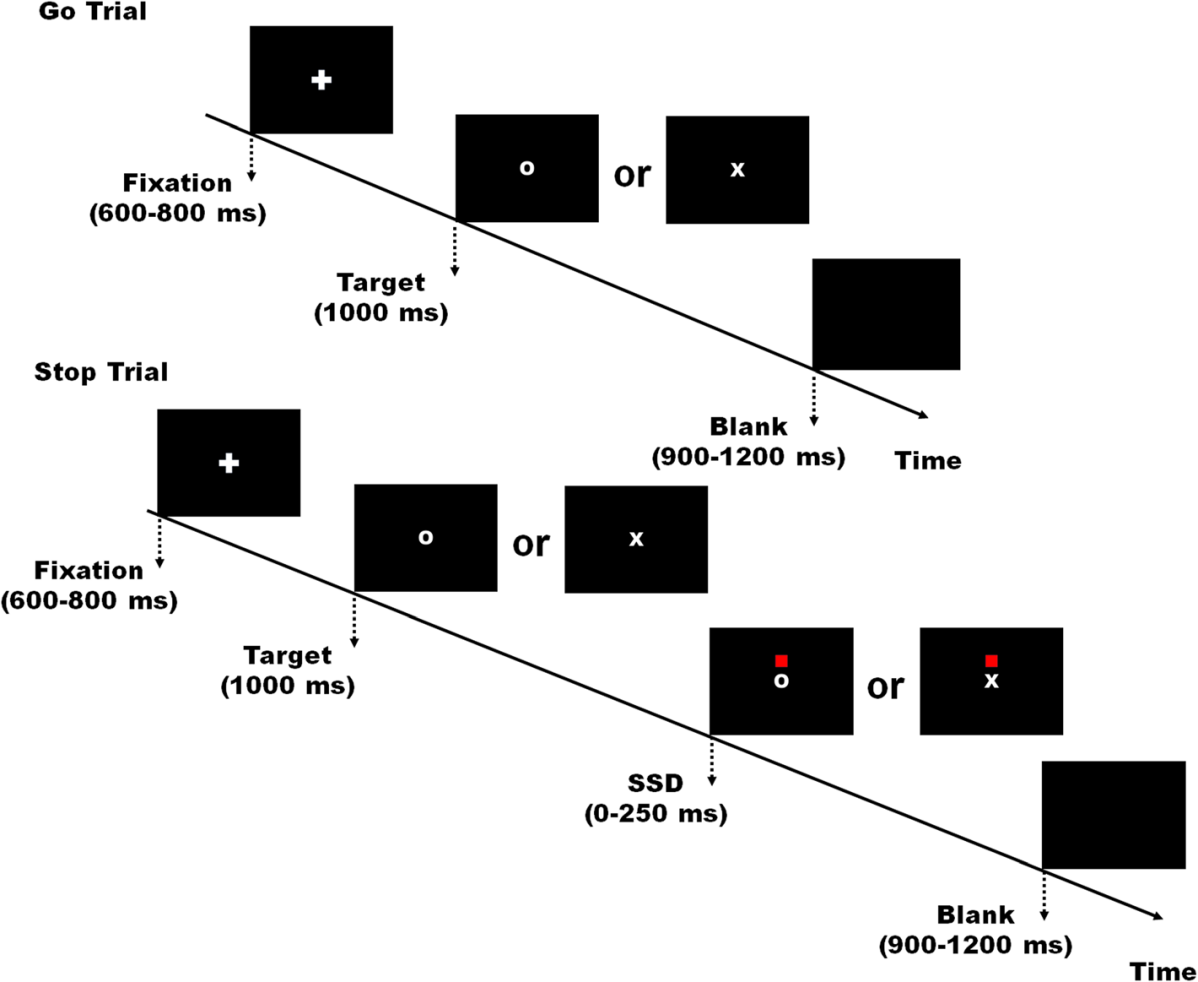
Stop signal paradigm. This task included 80% Go trials and 20% Stop trials. On Go trials, participants were presented with a fixation cross on a black computer screen lasting for 600–800 ms, which is immediately followed by a Go stimulus (the letter ‘X’ or ‘O’) (a Go signal) lasting for 1000 ms. Participants were instructed to judge the shape of the Go stimulus as accurately and quickly as possible via a button press with the index fingers of the left and right hands. On the remaining Stop trials (20%), the stop stimulus (a red square appearing above the location of the go stimulus) appeared after the Go stimulus after a variable delay of 0–250 ms in a step of 50 ms (the stop signal delay; SSD), cuing participants to withhold their responses to the Go stimulus. A variable intertrial interval was 1500–2000 ms.

We used the SST to measure response inhibition of ongoing actions, which is similar to that described in previous studies [33, 43]. The SST included 80% Go trials (320 trials) and 20% Stop trials (80 trials) (Fig. 1). On Go trials, participants were initially presented with a fixation cross on a black computer screen (600–800 ms), immediately followed by a visual stimulus (letter ‘X’ or ‘O’) indicating a Go signal (1000 ms). Participants were instructed to judge the shape of the visual stimulus as accurately and quickly as possible via a button press with the index fingers of the left and right hands (counterbalanced across participants). On Stop trials, the stop stimulus (a red square above the location of the go stimulus) appeared after the Go stimulus with a variable delay of 0–250 ms in steps of 50 ms (stop signal delay; SSD), cuing participants to withhold their responses to the Go stimulus. A blank screen followed each trial for a variable inter-trial interval (1500–2000 ms). Four experimental blocks were performed with a total of 400 trials. The experiment was preceded by a short practice block (20 trials). Task presentation was controlled via E-prime 2.0.

### EEG data recording and analysis

EEG data recording procedure was similar to that described in our previous studies [44, 45]. In brief, EEG was recorded (SynAmps amplifier, NeuroScan) with a quick cap carrying 64 Ag/AgCl electrodes placed at standard locations covering the whole scalp (the extended international 10–20 system). The reference electrode was attached to the right mastoid (M2), and the ground electrode was placed on the forehead. The vertical electrooculogram (VEOG) was recorded with electrodes placed above and below the left eye. The horizontal electrooculogram (HEOG) was recorded with electrodes placed beside the two eyes. The impedance was kept below 5 kΩ. The electrophysiological data were continuously recorded with a bandwidth 0.05–100 Hz and sampled at a rate of 1000 Hz.

Offline time-domain EEG data analysis was conducted using EEGLAB [46] and ERPLAB [47]. Data was first re-referenced to linked mastoid (M1 and M2). Independent component analysis (ICA)-based artifact correction was done by using the ICA function of EEGLAB. Saccades, blinks, and heart rate artifact were removed according to published guidelines [48]. The resultant EEG data were then epoched from 200 ms pre-stimulus to 1000 ms post-stimulus and digitally low pass filtered by 30 Hz (24 dB/octave). The 200 ms pre-stimulus period was used for baseline correction. In order to remove movement artifacts, epochs were rejected when fluctuations in potential values exceeded ±75 μV at any channels except the EOG channel. The ERPs were averaged separately for successful Stop trials and correct Go trials in each group.

Our time-frequency analysis was performed using the Matlab FieldTrip toolbox [49] using procedures as described in a recent study [50]. The EEG filtered data between 0.5–30 Hz was segmented 500 ms pre-stimulus onset to 1000 ms post-stimulus onset separately for Go and Stop trials per each group. Total event-related spectral power was obtained by transforming each epoch into the frequency domain using a sequential and overlapping unique Hanning window of 250 ms in steps of 25 ms with the multitaper time-frequency transformation (MTMCONVOL from ft_freqanalysis Fieldtrip software) method. In addition, the convolution function includes a ‘Zero’ type padding in order to cope with edging effects. After the transformation, we obtained a time-frequency spectrum with 1 Hz and 250 ms resolution. At each frequency, the results employed a dB transform [dB power = 10*log10 (power/baseline)] and were baseline corrected by subtracting the average baseline period (from − 200 to − 0 ms) from each data point. The obtained power values were then averaged over EEG epochs for trial types (i.e., Go and successful Stop trials) in each participant. Then, data were grand-averaged across MwoA patients and across healthy controls for each trial type.

### Statistical analysis

Demographic data were compared with non-parametric chi-square tests to assess between-group differences in sex ratio. Independent sample t-tests were used to examine between-group differences in age, years of education, anxiety (self-rating anxiety scale, SAS) and depression (self-rating depresson scale, SDS) and body mass index (BMI). The main variables analyzed with independent samples t-tests on the behavioral data were accuracy on Go trials (Go ACC), reaction times to Go stimuli (Go RTs) and the Stop signal reaction times (SSRTs).

Regarding statistical analysis on electrophysiological data, our data were analyzed according to the topographical distribution of grand averaged ERP activity as well as methods implemented in previous ERP studies [3, 38, 40, 51]. The ERP statistical analysis involved two ERP indices of response inhibition: the stop-N2 and stop-P3. Mean amplitudes for the stop-N2 (time interval = 200–250 ms, at the C3, Cz, C4, CP3, CPz, CP4, P3, Pz, P4 electrodes) and the stop-P3 (time interval = 350–500 ms, at the FC3, FCz, FC4, C3, Cz, C4, CP3, CPz, CP4, P3, Pz, P4 electrodes) were calculated. In order to examine effects of migraine on these ERP components, we conducted a mixed analysis of variance (ANOVA), with group as a between-participants factor (patients with MwoA versus healthy controls), and trial type (Go versus Stop trials), laterality (left [C3, CP3, P3], midline [Cz, CPz, Pz], right [C4, CP4, P4] for the N2; left [FC3, C3, CP3, P3], midline [FCz, Cz, CPz, Pz], right [FC4, C4, CP4, P4] for the P3) and area (central [C3, Cz, C4], centro-parietal [CP3, CPz, CP4], parietal [P3, Pz, P4] for the N2;fronto-central [FC3, FCz, FC4], central [C3, Cz, C4], centro-parietal [CP3, CPz, CP4], parietal [P3, Pz, P4] for the P3) as within-participants factors. Moreover, correlational analyses were performed to explore the potential relationship between the amplitude of the stop-N2 and the stop-P3 and clinical variables in Table 1. In addition, consistent with previous findings showing that delta and theta power accounts for activity underlying the stop-N2 and stop-P3 components in the stop signal task [38], the same statistical analyses were conducted. Based on visual inspection of time-frequency plots and the methods of previous studies [50–52], same area and laterality factors were included in such time-frequency analyses and mean power values in delta (1–4 Hz) and theta (4–8 Hz) frequency bands. The stop-N2 and stop-P3 components were extracted in selected time windows in order to disentangle the multiple processes underlying the stop N2–P3 complex associated to response inhibition.

All data were analyzed using IBM SPSS 19.0 (IBM Corp., Armonk, NY, USA). Statistical comparisons were made at *p*-values of *p* < 0.05, with the Greenhouse–Geisser correction when violations of sphericity occurred.

## Results

### Participant demographic

There were no statistically significant differences in age, sex ratio, education years, BMI, SAS, and SDS scores between patients with MwoA and healthy controls (Table 1).

### Behavioral results

A significant effect of group on Go RTs was found (t (45) = − 2.26, *p* < 0.05), indicating that patiens with MwoA (448.56 ms ± 40.28) responded more slowly than healthy controls (424.19 ms ± 33.71) on Go trials. Moreover, a significant effect of group on SSRTs was also observed (t (45) = − 2.30, *p* < 0.05), with SSRTs being longer in patients with MwoA (303.48 ms ± 41.87) than in healthy controls (278.44 ms ± 32.65). Similar levels of accuracy were found between groups on Go ACC (t (45) = 1.11, *p* = 0.27).

### Electrophysiological results

#### N2 (200–250 ms)

**Fig. 2:**
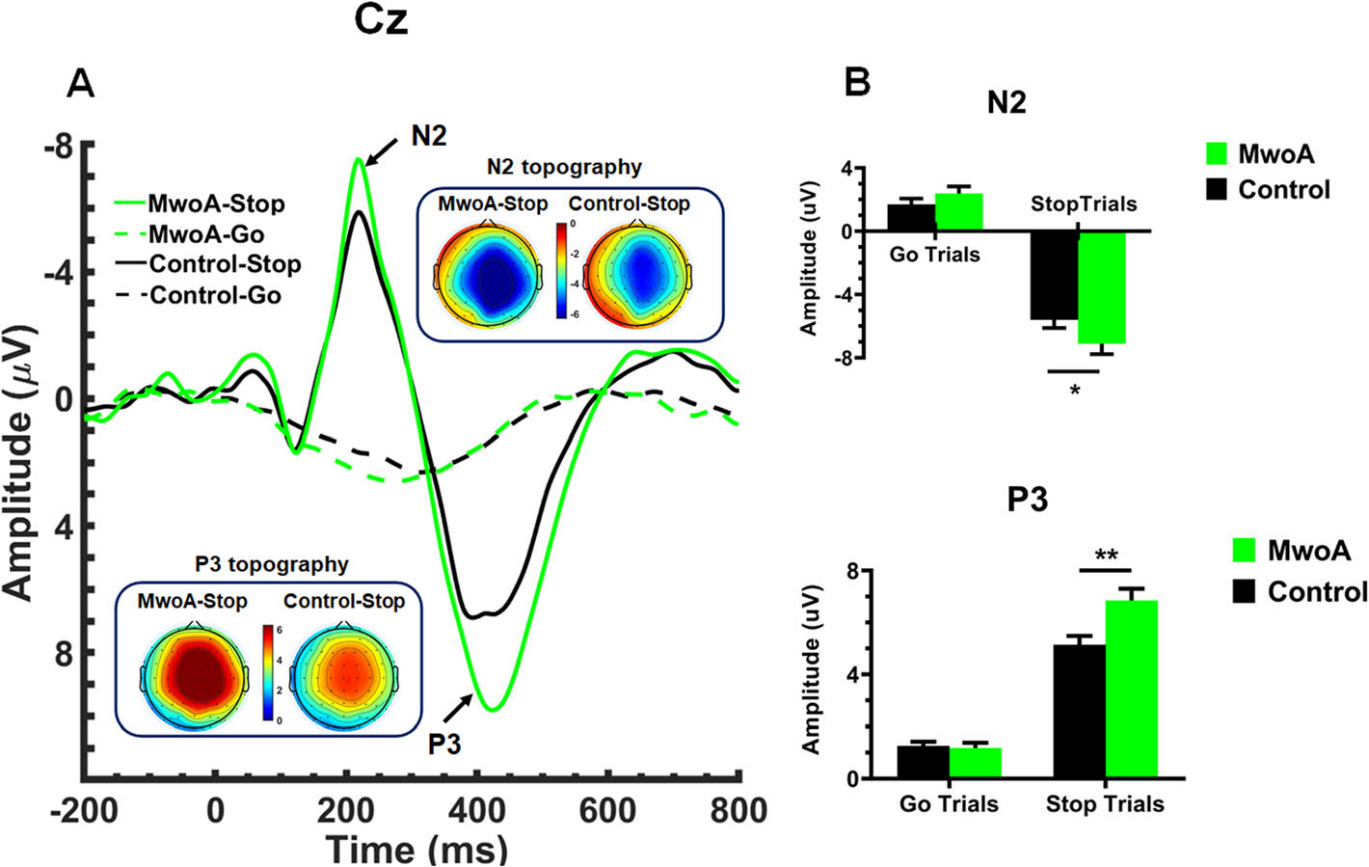
Time-domain ERPs results. **a** Grand average ERP waveforms recorded at Cz evoked by Go signals (dashed lines) and successful Stop signals (solid lines), and the topography of the N2 (200–250 ms) and P3 (350–500 ms) in patients with MwoA and healthy controls. **b** Means and standard errors (SEs) of the amplitudes of the N2 and P3 in the two groups. *denotes *p* < 0.05 and **denotes *p* < 0.01

A mixed ANOVA performed on the mean amplitude of N2 revealed a significant main effect of trial type (F (1, 45) = 383.95, *p* < 0.001), with the N2 amplitude being larger on Stop trials than on Go trials (*p* < 0.001). Moreover, a significant group X trial type interaction was found (F (1, 45) = 6.23, *p* < 0.05), due to larger amplitude of the N2 on Stop trials for patients with MwoA compared to healthy controls (*p* < 0.05) (Fig. 2). In addition, a significant main effect of area was also found (F(2,90) = 8.00, *p* < 0.005), quantified by a significant area X trial type interaction (F(2,90) = 7.53, *p* < 0.005). An analysis of simple effects revealed larger amplitudes of N2 in the central (*p* < 0.05) and centro-parietal regions (*p* < 0.005) than in the parietal region on Stop trials, while larger amplitude of the N2 in the central region than in the centro-parietal region (*p* < 0.001) on Go trials. Similarly, a significant main effect of laterality was further observed (F(2,90) =53.87, *p* < 0.001), quantified by a significant laterality X trial type interaction (F(2,90) = 83.26, *p* < 0.001), indicating larger amplitude of the N2 in the electrodes at the midline than that on the left (*p* < 0.001) and on the right (*p* < 0.001) on Stop trials and larger amplitude of the N2 in the electrodes on the right than that on the left on Stop trials (*p* < 0.001). No other significant effects were found. To explore the potential relationship between the amplitude of the stop-N2 and clinical variables in Table 1, our correlational analyses revealed a positive correlation between the amplitude of the stop-N2 in the central region and migraine duration in patients with MwoA (*r* = 0.49, *p* = 0.02).

#### P3 (350–500 ms)

Regarding the P3, a significant main effect of both group (F (1, 45) = 6.26, *p* < 0.05) and trial type (F (1, 45) = 330.19, *p* < 0.001) was found. Moreover, a significant group X trial type interaction was also found (F (1, 45) = 9.74, *p* < 0.005), indicating larger amplitude of the P3 in patients with MwoA than in healthy controls on Stop trials (*p* < 0.005) (Fig. 2). In addition, a significant main effect of area (F(3,135) = 25.74, *p* < 0.001) and a significant area X trial type interaction were found (F(3,135) = 32.58, *p* < 0.001), showing an increased P3 amplitude over the fronto-central (*p* < 0.001), central (*p* < 0.001) and centro-parietal regions (*p* < 0.001) compared to the parietal region on Stop trials, but an increased P3 amplitude in the centro-parietal region compared to other regions (all *p* < 0.005) on Go trials. Similarly, a significant main effect of laterality was found (F(2,90) = 71.18, *p* < 0.001), quantified by a significant laterality X trial type interaction (F(3,135) = 81.03, *p* < 0.001). The simple effects analysis showed larger amplitude of the P3 in the electrodes at the midline compared to the left and right electrodes on Go trials (all *p* < 0.05). However, larger P3 amplitude were seen at the midline than that in the electrodes on the left and on the right on Stop trials (all *p* < 0.001) and in the electrodes on the left than that on the right (*p* < 0.001) on Stop trials. No other significant effects were found. Correlational analyses were performed to identify the potential relationship between the amplitude of the stop-P3 and clinical variables in Table 1. We did not found any significant relationship between them in patients with MwoA (migraine duration: *r* = − 0.23, *p* = 0.32; history of migraine: *r* = 0.10, *p* = 0.65; migraine frequency: *r* = 0.11, *p* = 0.64; severity of headache: *r* = 0.10, *p* = 0.65).

### Time-frequency results

#### 200–250 Ms

Our mixed ANOVA analysis on theta power (4 ~ 8 Hz) revealed a significant main effect of trial type (F (1, 45) = 209.03, *p* < 0.001), with theta activity being larger on Stop trials than on Go trials (*p* < 0.001). There was also a significant main effect of area (F(2,90) = 16.71, *p* < 0.001), showing increased theta activity in the theta band in the central (*p* < 0.005) and centro-parietal regions (*p* < 0.001) relative to the parietal region. Similarly, there was a significant main effect of laterality (F(2,90) = 52.93, *p* < 0.001), quantified by a significant laterality X trial type interaction (F(2,90) = 33.40, *p* < 0.001). Our simple effects analysis revealed increased theta activity at the midline compared to the electrodes on the left (*p* < 0.001) and on the right (*p* < 0.001) on Go trials. Meanwhile, increased theta activity at the midline relative to the electrodes on the left (*p* < 0.001) and on the right (*p* < 0.001) on Stop trials was found and increased theta activity in the electrodes on the left compared to that on the right on Stop trials (*p* < 0.001) was also found. No other significant effects were found.

Furthermore, delta power (1 ~ 4 Hz) effects showed a significant main effect of trial type (F (1, 45) = 97.00, *p* < 0.001), indicating increased spectral power in the theta frequency band on Stop trials compared to Go trials (*p* < 0.001). In addition, a significant main effect of area was also found (F(2,90) = 7.92, *p* < 0.005), quantified by a significant area X trial type interaction (F(2,90) = 16.28, *p* < 0.001). The simple effects analysis revealed increased delta activity in the centro-parietal region compared to the central (*p* < 0.001) and parietal regions (*p* < 0.05) on Go trials. Moreover, increased delta activity in central (*p* < 0.05) and centro-parietal regions (*p* < 0.005) was observed compared to the parietal region in Stop trials (*p* < 0.001). A significant main effect of laterality was found (F(2,90) = 38.35, *p* < 0.001), quantified by a significant laterality X trial type interaction (F(2,90) = 41.69, *p* < 0.001). The analysis of simple effects further revealed increased delta activity at the midline compared to the electrodes on the left (*p* < 0.01) and the right (*p* < 0.001) on Go trials. Increased delta activity at the midline relative to the electrodes on the left (*p* < 0.001) and on the right (*p* < 0.005) on Stop trials was found and increased delta activity in the electrodes on the right relative to that on the left (*p* < 0.001) on Stop trials was observed. No other significant effects were found.

#### 350–500 Ms

**Fig. 3:**
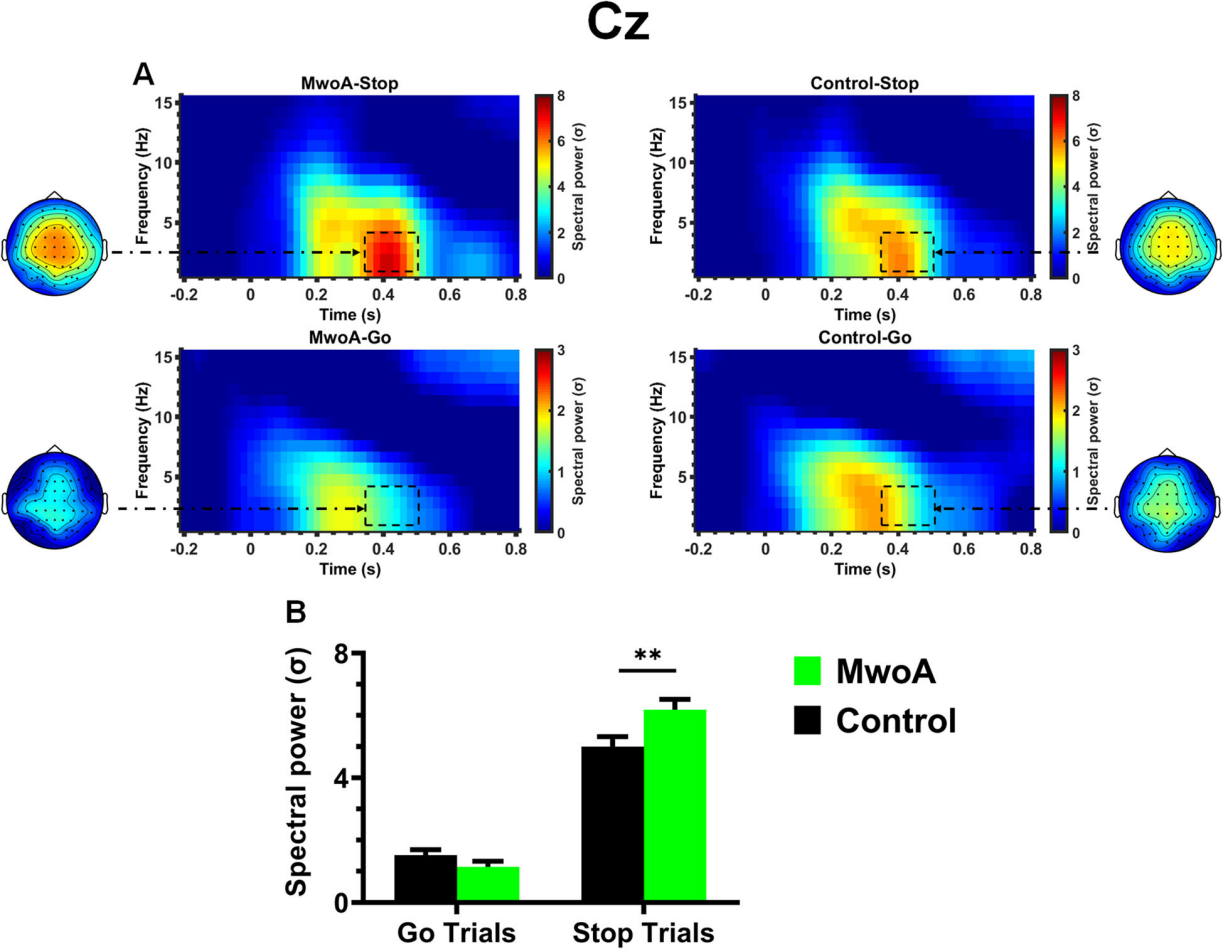
Time-frequency results. **a** Time-frequency plots showing delta changes in normalized power in go and stop trials (left) for patients with MwoA and healthy controls for the selected electrode (Cz). Black dotted squares indicate the time-frequency region in which theta power in stop trials was significantly higher in patients with MwoA than in healthy controls. Scalp topography maps show the spatial distribution of theta power (4–8 Hz) in go and stop trials between 350 and 500 ms. The color scale indicates spectral power in SD. **b** Means and standard errors (SEs) of the delta power (4–8 Hz) between 350 and 500 ms at Cz in the two groups. **, *p* < 0.01

The mixed ANOVA analysis on delta power (1 ~ 4 Hz) showed a significant main effect of trial type (F (1, 45) = 306.97, *p* < 0.001), showing increased spectral power in the delta band on Stop trials compared to Go trials (*p* < 0.001). Moreover, a significant group X trial type interaction was found (F (1, 45) = 9.42, *p* < 0.005), due to delta increased activity on Stop trials for patients with MwoA compared to healthy controls (*p* < 0.05) (Fig. 3). In addition, a significant main effect of area was also observed (F(3,135) = 10.92, *p* < 0.001), quantified by a significant area X trial type interaction (F(3,135) = 23.86, *p* < 0.001), indicating larger delta activity in the centro-parietal region than in the central region on Go trials (*p* < 0.05), but stronger delta activity in the fronto-central (*p* < 0.005), central (*p* < 0.001) and centro-parietal regions (*p* < 0.001) than in the parietal region on Stop trials. Similarly, a significant main effect of laterality was observed (F(2,90) = 35.16, *p* < 0.001), quantified by a significant laterality X trial type interaction (F(2,90) = 19.64, *p* < 0.001), showing that delta activity reached its maximum in the midline electrodes compared with the left (*p* < 0.001) and right electrodes (*p* < 0.001) on Go trials, whereas delta activity on Stop trials was stronger in the midline electrodes than on the left (*p* < 0.001) and right electrodes (*p* < 0.001) and was also larger on the right electrodes than on the left ones (*p* < 0.05). No other significant effects were found.

Regarding theta power (4 ~ 8 Hz), a significant main effect of trial type was observed (F (1, 45) = 213.19, *p* < 0.001), showing increased spectral power in the theta band on Stop trials compared to Go trials (*p* < 0.001). Moreover, a significant main effect of area was also found (F(3,135) = 32.24, *p* < 0.001), quantified by a significant area X trial type interaction (F(3,135) = 37.21, *p* < 0.001), indicating stronger theta activity in the centro-parietal region than in the parietal region on Go trials (*p* < 0.01), whereas larger theta activity in the fronto-central, central and centro-parietal regions than in the parietal region on Stop trials (all, *p* < 0.001). Similarly, a significant main effect of laterality was observed (F(2,90) = 47.18, *p* < 0.001), quantified by a significant laterality X trial type interaction (F(3,135) = 21.65, *p* < 0.001). Our simple effects analysis showed that theta reached its maximum activity in the midline electrodes than on the left (*p* < 0.001) and on the right ones (*p* < 0.001) on Go trials. In contrast, on Stop trials, theta activity was stronger in the midline electrodes than on the left (*p* < 0.001) and on the right electrodes (*p* < 0.001) and on the right electrodes than on the left ones (*p* < 0.001). No other significant effects were found.

## Discussion

To the best of our knowledge, the present study is the first to investigate the pathophysiological features of response inhibition during the interictal period of patients with MwoA. Consistent with the theory that alterations in cognitive cortical processes is a key signature of migraine [21, 47, 53], the results here revealed an abnormal state of suppressing prepotent responses in migraineurs attributable to functional cortical disexcitability. Our results imply the existence of dysfunctional inhibitory control at later stages of the neurophysiological spectrum during information processing.

At the behavioral level, patients with MwoA relative to healthy controls showed significantly slower inhibition speed (SSRTs). Given that the SSRTs provide an effective means of quantifying the latency of the inhibition mechanism, the slower SSRTs implied a hyperexcitable state of response inhibition in migraineurs. Reduced performance during a stop signal task is in line with previous studies on other dimensions of inhibition control, showing slower RTs on incongruent trials during the Stroop interference test but without between-group differences in errors [32, 54]. Given that previous research has reported inhibition control deficits during the early and intermediate stage of information processing in migraineurs [32, 55], this study expands current knowledge of migraineurs’ dysfuntional inhibition control by showing the existence of altered responses inhibition in migraineurs. Furthermore, prior evidence has revealed a significant drop in several aspects of cognitive functioning in migraineurs, including sustained attention/concentration, working memory and executive function, during migraine attacks [29, 56–58], during its chronicity [31, 59–61] and during the intervals between attacks [53, 62, 63]. Therefore, it may be plausible to suggest that the abnormal response inhibition hereby reported may not only reflect a dysfunctional inhibition control by itself, but may also be driven by a decrease in overall cognitive efficiency in patients with MwoA.

At the neural level, consistent with the notion that cortical disexcitability is a key signature of migraine [21, 47, 53], we first found that patients with MwoA relative to healthy controls were characterized by a pronounced increase in the N2 amplitude on stop trials in central and parieto-central regions. In spite of some controversy about its functional significance, the stop-N2 is proposed to be related to conflict monitoring that is involved in the initial detection of conflict between response execution and inhibition [38, 39, 64]. As a consequence, the increased stop-N2 amplitude seems to reflect a hyperexcitable state of conflict monitoring that controls the concurrent and competing co-activation of response representations during early, non-motoric stages of response inhibition in patients with MwoA. Despite such findings on the stop-N2 amplitude, no difference was seen in the topographical distribution of this ERP index between patients and controls. The stop-N2 reached its maximum in the central and centro-parietal regions across both groups. Considering source-localization studies report the neural generator of the stop-N2 is located around the anterior cingulate cortex (ACC) [38, 64–67], this seems to suggest that the presence of deficient inhibitory cortical processes can be localized beyond the sensory cortical system in the migraine brain. Although our observation of increased stop-N2 amplitude provides compelling evidence in support of a hyperexcitable state of conflict monitoring during early, non-motoric stages of response inhibition, this cannot answer the question of whether other underying processes involved in response inhibition are altered. Further analysis on the amplitude of the P3 additionally contributes to addressing this issue, since patients exhibited an increase in the stop-P3 amplitude along fronto-central, central and centro-parietal regions. Contrary to the stop-N2, this ERP component has been proposed to reflect late-stage inhibition of the motor system itself and cognitive evaluation of motor inhibition [38]. As such, it is also possible to claim that the increased stop-P3 amplitude seen in patients with MwoA indicates a hyperexcitable state of inhibition control during the late stage of response inhibition (when inhibiting a motor response). Given that source-localization studies have also shown that stop-P3 is mainly associated with neural activity of the primary motor cortex (M1) and the supplementary motor areas (SMA) [38, 68], this therefore may reflect dysfunctional activity in these cortical regions and circuits in migraineurs.

Taken together, the time-domain ERP results during a stop signal task are suggestive of neurocognitive deficits while suppressing a prepotent motor response as evidenced by the increased ERP indices of response inhbitionn (stop N2–P3 complex) in patients with MwoA relative to healthy controls. Although the arguments above focus on the maladaptive inhibitory circuit in patients with MwoA, this does not rule out alternative accounts for pathophysiological features of dysfunctional response inhibition in migraineurs. Given that response inhibition is an important component of the executive system [69, 70] and weaker functional connectivity within regions of the pre-frontal executive network (middle frontal gyrus and dorsal anterior cingulate cortex) has been well documented [18, 20, 71], there is a high probability that weaker network activity within the pre-frontal executive regions may lead to dysfunctional response inhibition in patients with MwoA. In addition, sensory and motor networks in the centro-parietal regions have been found to show altered long-range functional connections to higher order networks [18, 72]. This may indicate that alterations in network activity within centro-parietal sensorimotor regions and weaker long-range network connections to higher order regions may additionally lead to the pathophysiological features of response inhibition in patients with MwoA.

The higher expression of ERPs components associated with response inhibition over fronto-central, central and centro-parietal areas are in agreement with previous findings showing a lack of habituation and overweight of cortical excitatory vs. inhibitory processes in migraine patients [12, 55]. Moreover, the results may also have important clinical implications. Over the past decade, much progress has been made in the understanding of migraine pathophysiology. Our electrophysiological findings may serve as useful clinical biomarkers for identifiying response inhibition dysfunction in migraine patients, thereby contributing to a greater appreciation of physiological features of inhibitory control processes in migraine patients. Improved characterisation and diagnosis of clinical features based on the pathophysiological characteristics of inhibition control may lead to novel targets for migraine therapy and provide new opportunities for more effective patients’ management of patients and their relatives.

Although these two time-domain ERP indices of response inhibition greatly contribute to our understanding of how the brain detects the stop signal and decides to stop a prepotent motor response in patients with MwoA, ERP measures cannot accurately reflect the overlapping and underlying processes underlying response inhibition. To address this issue, we characterized event-related theta (4–8 Hz) and delta (1–4 Hz) oscillations to index two separable but highly overlapping processes underlying the stop N2–P3 complex in response inhibition. Time-frequency analysis found significantly higher theta and delta activities on stop trials (compared to Go trials) in the time windows used to extract mean amplitudes of the stop-N2 and stop-P3 components, which is consistent with findings from previous studies [38, 40, 50, 51]. Although both theta and delta activities in response to either the stop signal or Go stimuli in the time window used to extract mean amplitude of the stop-N2 component did not highlight any significant difference between groups, patients with MwoA showed larger delta activity (not theta activity) relative to healthy controls on stop trials associated with the stop-P3 component. Despite ongoing debates on the functional significance of these two oscillatory activities [38], the relationship of TF phase dynamics to time-domain ERP measures may substantially explain the time-domain experimental effects observed in the present study. Given that previous studies have found that delta activity relative to theta activity contributed more to the stop-P3 component [38, 42], this may suggest that increased time-domain stop-P3 observed in patients with MwoA relative to healthy controls is mainly driven by the delta activity in the same time window. In contrast to delta-related stop-P3 component, we failed to find an interaction effect between group and trial type on both theta and delta activities in the time window used to extract mean amplitude of the stop-N2. Since theta and delta oscillations have been suggested to contribute uniquely in the opposite direction at the N2, one possible explanation is that their combination may lead to the non-significant interaction effect between group and trial type in the frequency domain in the time time used to extract the mean amplitude of the stop-N2.

### Limitations

In spite of our significant findings, the present study still counts with some limitations. First, we included a relative small sample size, thus possibly tempering the strength of our conclusions. Hence, future studies should replicate our results with larger sample sizes. Second, the present study included patients suffering from migraine without aura. Given that migraine is a heterogeneous disease and the difference between migraine subtypes has been increasingly highlighted [73–75], this may affect “generalizability” of the results in migraineurs and therefore foster future research exploring the pathophysiology of response inhibition in other subforms of this disease. Third, although the stop signal task is a frequent measurement of response inhibition, there are other types of task that can be used to measure inhibitory control such as the Go/no-go task. Moreover, accumulating evidence has revealed differences between inhibitory measures, raising the question of whether they tap into equivalent cognitive mechanisms underlying inhibitory control capacities [76]. As a consequence, future research is required to examine other measures of response inhibition to better characterize the pathophysiological basis of inhibition control at later stages of information processing such as response execution/initiation. Last but not the least, recent studies have revealed that cortical excitability (e.g., the motor cortex) covaries with the time elapsed from the last attack in migraine patients [77, 78]. However, in the present study, the time elapsed since the last migraine attack were not collected, thus making it impossible to explore its relationship with the neurophysiological indices of response inhibition (the stop-N2 and stop-P3). Future studies should take it into account. Despite these limitations, we still believe that our findings are still robust and may foster further research on neuroanatomical characteristics and pathological signatures underlying inhibition control in migraineurs.

## Conclusions

The present study was designed to characterize pathophysiological features of response inhibition using event-related potentials (ERPs) in patients with MwoA during the interictal period. Our main finding is that patients with MwoA displayed prolonged SSRTs relative to healthy controls, indicating a decrease in inhibiting a prepotent response in migraineurs. At the brain level, the amplitudes of the stop-N2 and stop-P3 over the fronto-central, central and centro-parietal regions were significantly increased in patients with MwoA compared with healthy controls. Moreover, time-frequency decompositions have revealed increased delta activity in the time window used to extract to the mean amplitude of stop-P3 in patients with MwoA relative to healthy controls. These findings indicate a dysfunction in ERP indices of sub-processes underlying response inhibition in patients with MwoA, which can be attributable to cortical disexcitability. Therefore, the present study offers novel insights into how the brain detects and executes stopping behaviours in migraineurs. Ultimately, our findings also have important implications for stimulating future research characterizing inhibitory control alterations in migraineurs to optimize putative clinical interventions.

## Data Availability

The datasets used and analyzed during the present study are available from the corresponding authors on reasonable request.

## Abbreviations

MwoA: Migraine without aura
SST: Stop signal task
ERPs: Event-related potentials
SSRT: Stop signal reaction time
ACC: Anterior cingulate cortex
SMA: Supplementary motor area
